# Optimization of Plasma Activated Water Extraction of *Pleurotus ostreatus* Polysaccharides on Its Physiochemical and Biological Activity Using Response Surface Methodology

**DOI:** 10.3390/foods12234347

**Published:** 2023-12-01

**Authors:** Fuangfah Punthi, Bara Yudhistira, Mohsen Gavahian, Chao-Kai Chang, Naila Husnayain, Chih-Yao Hou, Cheng-Chia Yu, Chang-Wei Hsieh

**Affiliations:** 1 Department of Food Science and Biotechnology, National Chung Hsing University, Taichung City 40227, Taiwan; fuangfahp3@gmail.com (F.P.); barayudhistira@staff.uns.ac.id (B.Y.); kai70219@nchu.edu.tw (C.-K.C.); 2 Department of Food Science and Technology, Sebelas Maret University, Surakarta City 57126, Indonesia; 3 Department of Food Science, National Pingtung University of Science and Technology, Pingtung City 91201, Taiwan; mg@mail.npust.edu.tw; 4 International Master Program of Agriculture, National Chung Hsing University, Taichung City 40227, Taiwan; nailahusnayain@gmail.com; 5 Department of Seafood Science, National Kaohsiung University of Science and Technology, Kaohsiung City 81157, Taiwan; chihyaohou@nkust.edu.tw; 6 Institute of Oral Sciences, Chung Shan Medical University, Taichung City 40201, Taiwan; ccyu@csmu.edu.tw; 7 Department of Dentistry, Chung Shan Medical University Hospital, Taichung City 40201, Taiwan; 8 Department of Medical Research, China Medical University Hospital, Taichung City 40402, Taiwan

**Keywords:** *Pleurotus ostreatus*, plasma-activated water, extraction, polysaccharides, antioxidant activity, response surface methodology

## Abstract

This study focused on optimizing the extraction of *P. ostreatus* polysaccharides (POPs) using plasma-activated water (PAW). A single factor and response surface methodology were employed to optimize and evaluate the polysaccharide yield, physiochemical characteristics, and biological activities of POPs. The observed findings were compared to those obtained by the conventional hot water extraction method (100 °C, 3 h), as the control treatment. The optimal extraction conditions were obtained at 700 W PAW power, 58 s treatment time, 1:19 sample-to-water ratio, and 15 L/min gas flow rate. In these conditions, the PAW-treated samples experienced changes in surface morphology due to plasma etching, leading to a 288% increase in the polysaccharide yield (11.67%) compared to the control sample (3.01%). Furthermore, the PAW-treated sample exhibited superior performance in terms of biological activities, namely phenolic compounds (53.79 mg GAE/100 g), DPPH scavenging activity (72.77%), and OH scavenging activity (65.03%), which were 29%, 18%, and 38% higher than those of control sample, respectively. The results highlighted the importance of process optimization and provided new evidence for PAW as an alternative approach to enhance the extraction efficiency of POPs, a novel source of natural antioxidants which enables diverse applications in the food industry.

## 1. Introduction

An increase demand for natural-based products used in healthcare and wellness industries has prompted interest in utilizing plants for medicinal purposes as potential sources of functional and bioactive components, particularly in the aftermath of the recent epidemic [1]. 

Natural polysaccharides, which are nontoxic, biocompatible, biodegradable, widely available in plants, and exhibit high antioxidant activity, show promise in the treatment and prevention of diseases caused by oxidative stress [1]. They have a crucial therapeutic function and have gained popularity in recent decades due to their vital antioxidant, anticancer, antibacterial, and immunoregulatory properties [2,3,4]. Mushroom polysaccharides have gained increasing interest due to the presence of a variety of biological characteristics, including antioxidant properties, which are extensively employed in pharmaceutical, nutritional, cosmetic, and other industries [2,5].

*Pleurotus ostreatus* (*P. ostreatus*) stands as the most widely consumed edible mushroom globally. The cultivation of this mushroom has been regarded as economically significant due to its substantial production output and adaptability [6]. *P. ostreatus* polysaccharides (POPs) have attracted growing interest in recent years, owing not only to the delicacy of their flavor but also to being known as a nutritious food with various biological activities. These activities are antioxidants, anticancer, anti-inflammatory, immunomodulatory, and prebiotic functions [5,6]. A greater awareness of these polysaccharides’ health benefits could drive market demand for POPs in the nutraceutical and pharmaceutical sectors. The proclivity of the cosmetic industry toward natural and sustainable ingredients suggests the potential integration of POPs into skincare and beauty products. Staying informed about industry trends and current scientific research in this domain is imperative for a more comprehensive understanding of POP use [6,7]. With the advancement of extraction technology, the study of the active compounds and biological activities of *P. ostreatus* has become prominent in recent years [5]. The technique of extraction utilized for recovering plant matrix polysaccharides is critical for preserving their biological and quality potential. The biological potential of natural extracts depends on both the source of the plant matrix and the extraction method utilized [1]. 

POP primarily consists of water-soluble compounds that exhibit solubility in water at high temperatures [6]. Despite its simplicity, extracting these polysaccharides with hot water presents challenges, primarily due to inadequate penetration into the mushrooms. Therefore, there is a significant focus on the development and optimization of hot water extraction methods for mushroom polysaccharides in previous research. There is much research that explores alternative techniques, including alkaline and acid extraction, microwave-assisted extraction, and ultrasound-assisted extraction [6,8,9,10]. However, recent studies have shed light on the environmental drawbacks associated with these methods. Issues such as low extraction yield, extended extraction time, and the necessity for multiple extraction runs have been identified. These limitations have the potential to result in the degradation of polysaccharides and a reduction in the biological activity of the target compounds [4,5,9].

Cold plasma (CP) is a promising non-thermal technology with well-explained surface etching effects that can potentially enhance the mass transfer [11]. Such a concept can potentially increase the extraction yield (e.g., in phytochemical extraction). CP treatment allows compounds of interest to be released more efficiently and improve the extraction yield [12]. This technology aligns with the growing demand for environmental and economic sustainability, primarily due to its energy efficiency, enabling shorter extraction times, and minimizing the degradation of heat-sensitive substances [1]. Plasma-activated water (PAW) is an emerging form of CP that employs plasma devices for treating water, causing the formation of a solution rich in chemically reactive oxygen and nitrogen species (RONS) [13]. This enormous number of active species from chemical donors in an aqueous environment has a beneficial influence the enhanced permeability of active species in food and substantially alters the functioning of lipids, proteins, and carbohydrates in food. PAW allows for a more intense visualization of hydrogen bond rupturing, amino acid oxidation, and prosthetic group modifications [14]. RONS disrupt both cell membranes and intracellular parts, resulting in a breakdown of cell membranes. Moreover, plasma induces a strong electric field that gives rise to the formation of electropores inside cellular membranes, culminating in the release of cellular contents and the death of cells [15]. Presently, PAW has also demonstrated promising outcomes in several fields, including improving food shelf life, food contamination, starch modification, and food packaging characteristics [16,17,18]. Also, several plasma factors, including gas type, applied voltage, treatment time, and water-to-sample ratio, have been reported to affect the efficacy of plasma-induced extraction [13,19]. However, only a few studies have employed cold plasma technology for improving *P. ostreatus* germination and sterilization [20]. Additionally, there is a limitation in research on the impact of PAW treatment on plant extraction regarding its effect on phytochemicals and biological potential, notably in plant polysaccharide extraction [18].

The response surface methodology (RSM) is frequently utilized to optimize the condition parameters of bioactive compound extraction [3,21]. The RSM determines the interaction between multiple factors by creating the appropriate mathematical model. This approach requires fewer experimental trials, demonstrating its high significance [4]. To the best of our knowledge, there is a lack of research undertaken on the RSM-optimized PAW extraction of POP. Therefore, this study is the first to examine and optimize the PAW extraction of polysaccharides from *P. ostreatus* using the RSM. This study introduces a novel approach aimed at retrieving an extract replete with biological and antioxidant activities, thereby positioning it as a potential alternative natural source of polysaccharides. 

## 2. Materials and Methods

### 2.1. Mushroom Preparation

*P. ostreatus* was obtained from Q-Yo Bio-Technology Co., Ltd., Changhua, Taiwan. All *P. ostreatus* samples, with a moisture content of 88.72 ± 0.23% were washed on the obtained day and stored at −20 °C for 24 h before freeze drying for 48 h. Dried *P. ostreatus* was then crushed into powder using a grinder (Oster, Milwaukee, WI, USA), passed through a mesh sieve and examined for moisture content (6.15 ± 0.05%) before being stored for further polysaccharide extraction. 

### 2.2. Chemical and Reagents

L(+)-Ascorbic acid and Folin-Ciocalteu’s reagent were purchased from Panreac Applichem, Chicago, IL, USA. In addition, 2,2-diphenyl-1-picrylhydrazyl (DPPH), 2,2-Azino-bis(3-ethylbenzo-thiazoline-6-sulfonic acid) diammonium salt (ABTS), Iron(II) sulfate heptahydrate, and D-(+)-Glucose was purchased from Sigma Co., Saint Louis, MO, USA. Potassium persulfate, hydrogen peroxide (30%), sulfuric acid, and sodium carbonate anhydrous were purchased from KATAMAYA (Osaka, Japan). Sodium salicylate and phenol were purchased from Daejung (Siheung-si, Republic of Korea), while gallic acid monohydrate was purchased from Scharlau (Barcelona, Spain).

### 2.3. Plasma-Activated Water (PAW) Extraction

**Figure 1:**
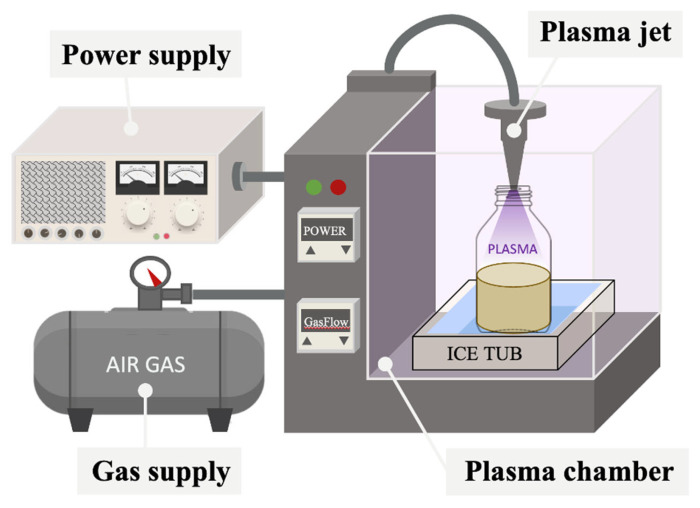
Schematic representation of the PAW treatment system.

**Figure 2:**
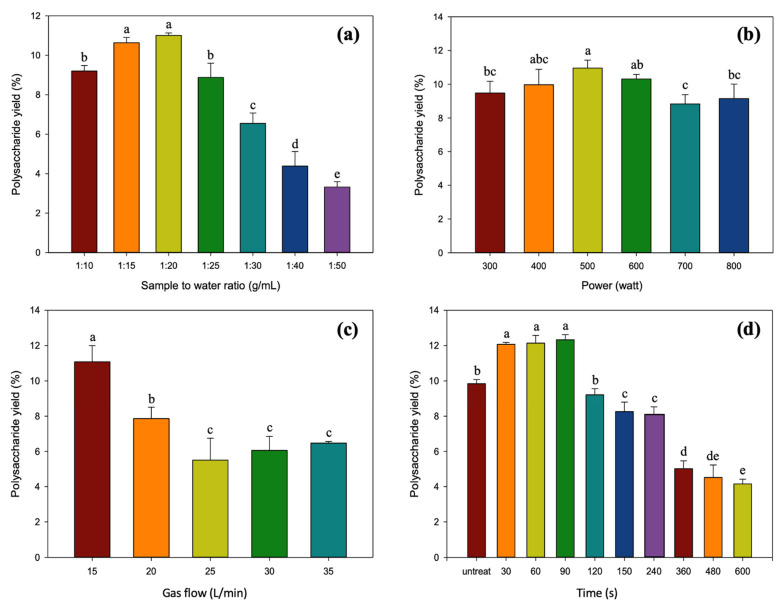
Effects of different PAW parameters on the POP extraction yield. (**a**) Sample-to-water ratio (fixed as 400 W of power, 20 L/min of gas flow, and 3 min of treatment time); (**b**) power (fixed as 1:20 sample-to-water ratio, 20 L/min of gas flow, and 3 min of treatment time); (**c**) gas flow (fixed as 1:20 sample-to-water ratio, 400 W of power, and 3 min of treatment time); and (**d**) treatment time (fixed as 1:20 sample-to-water ratio, 400 W of power, and 20 L/min of gas flow). ^a–e^ The values with different superscripts exhibit statistical significance (*p* < 0.05). The error bars in the graph represent the standard deviations (*n* = 3).

The PAW device was designed and constructed by the Metal Industries Research & Development Center, Kaohsiung, Taiwan which consists of a plasma generator, a 4 × 5 × 4 m^3^ plasma chamber, a power supply, and gas supply (Figure 1). The PAW employed in this study was implemented using a non-thermal atmospheric pressure plasma jet (APPJ) generator in a chamber at room temperature (25 °C), utilizing air as the gas supply. For the PAW extraction treatment, a 500 mL borosilicate glass bottle filled with 200 mL of the mixed solution (*P. ostreatus* powder and distilled water). An APPJ electrode was positioned 10 cm above the mixed sample surface and plasma jet nozzle. For preliminary studies, the sample-to-water ratio solution, the input power, the gas flow rate, and the PAW treatment time were equipped in varying conditions, presented in Figure 2. Importantly, the sample solution was maintained at room temperature using a 9 m^3^ ice tub.

The crude polysaccharides was extracted using Hsiao et al. [22] modified method. In brief, after treatment of PAW, the extracted sample was filtered using a vacuum filtration machine (Tokyo Rikakikai, Tokyo, Japan) with Whatman filter paper No. 1. Then, it was precipitated by the addition of a 2-fold volume of absolute ethanol and thereafter stored at 4 °C. Subsequently, the obtained precipitate was centrifuged (Hitachi CR22N, Thermo Fisher Scientific, Waltham, MA, USA) at 7155× *g* for 15 min, then the supernatant was washed with diethyl ether and achieved by lyophilizing (Kingmech, New Taipei City, Taiwan) for 48 h to obtain the crude *P. ostreatus* polysaccharides. The production yield of crude polysaccharide was determined and obtained sample was stored for further analysis. The polysaccharide yield (%) was calculated as follows:

Polysaccharide yield (%, *w*/*w*) = (weight of total polysaccharide (g)/weight of dried mushroom powder (g)) × 100 (1)

### 2.4. Hot Water Extraction

In this study, hot water extraction was conducted to compare the efficiency of extraction with the PAW extraction method, using the modified of Barbosa et al. [9] method. Initially, 10 g of dried *P. ostreatus* powder was mixed with 200 mL of distilled water and then extracted using a water bath at 100 °C for 3 h, with a sample-to-water ratio of 1:20. Subsequently, the mixture was filtered, alcohol precipitated, the supernatant collected, and the following extraction step was performed in accordance with Section 2.3. The obtained sample was then analyzed for polysaccharide yield and stored for further investigations.

### 2.5. Experimental Design

Single-factor experimentation and the RSM are both methodologies used in experimental design to optimize extraction conditions [23,24,25]. Single-factor experimentation is an approach that examines how individual factors affect a process while keeping other factors constant [24]. It aids in identifying the critical factors that significantly influence the extraction. However, it does not account for potential interactions between factors which might not reveal the optimal combination of factor levels. The RSM is a statistical and mathematical method for optimizing complicated processes by examining the relationship between multiple independent variables and the response [26], providing a more realistic representation. In this study, we conducted a combination of single-factor analysis and the RSM optimization to systematically investigate the parameters for POP extraction. Our goal was to efficiently identify optimal conditions and gain insights into the intricate relationships among factors influencing the extraction process.

#### 2.5.1. Single Factor Analysis

A single factor analysis of the influence of PAW extraction on POP yield was determined in accordance with the operational capacity of the PAW machine in use, supplemented by a review of relevant literature that has been documented as effective and suitable. For this study, the following four variables were investigated: (1) sample-to-water ratio of 1:10, 1:15, 1:20, 1:25, 1:30, 1:40, and 1:50 g/mL; (2) power of 300, 400, 500, 600, 700, and 800 W; (3) gas flow of 15, 20, 25, 30, and 35 L/min; and (4) treatment time of 30, 60, 90, 120, 150, 240, 360, 480, and 600 s. The suitable range of each variable obtained in high polysaccharide yield was selected for further optimization study.

#### 2.5.2. Optimization Using the RSM

The key aspect of the RSM experimental design is establishing factor boundaries. Based on the findings of the single-factor analysis, the appropriate ranges for three independent variables were selected for the RSM study: sample-to-water ratio (g/mL) with a fixed total volume of the mixture at 200 mL, power (W), and treatment time (s). These factors were then converted into coded units, ranging from −1 to +1. The coded representation of extraction conditions is delineated as follows: the sample-to-water ratio (X_1_) ranged from 1:10–1:30 g/mL, power (X_2_) from 300 to 700 W, and time (X_3_) from 30 to 150 s, while maintaining a constant gas flow at 15 g/mL. For the RSM analysis, a three-variable, three-level Box–Behnken design was employed to optimize the processing parameters for the extraction of POPs. This optimization was directed towards achieving maximum yield of polysaccharides (%) and antioxidant activities (DPPH, ABTS, and OH assay). The experimental design resulted in a total of 15 runs, as presented in Table 1. The optimal extraction variables of the responses were determined by calculating the following second order polynomial formula:

(2) $$Y=A_{0}+A_{1}X_{1}+A_{2}X_{2}+A_{3}X_{3}+A_{4}X_{1}^{2}+A_{5}X_{2}^{2}+A_{6}X_{3}^{2}+A_{7}X_{1}X_{2}+A_{8}X_{1}X_{3}+A_{9}X_{2}X_{3}$$

where Y represents dependent variable, and the estimated regression coefficients of the independent variables for the intercept (A_0_), the linear coefficients (A_1_, A_2_, and A_3_), the quadratic coefficients (A_4_, A_5_, and A_6_), and interaction coefficients (A_7_, A_8_, and A_9_). To optimize the extraction conditions of POP, the RSM was employed, and the coefficients of the polynomial model and optimization were calculated using the Minitab Version 21 software (Minitab, LLC, State College, PA, USA). After determining the optimum variables for PAW extraction, the *P. ostreatus* sample was subjected to the optimal PAW extraction conditions to confirm. The acquired results were compared in terms of physiochemical characteristics and biological activities to those of an unextracted (*P. ostreatus* powder) sample and a hot water extraction sample.

### 2.6. Chemical Analysis

#### 2.6.1. Antioxidant Activity

DPPH radical scavenging activity, ABTS radical scavenging activity, and hydroxyl radical (OH) scavenging activity were carried out in accordance with the modified methodology of Gąsecka et al. [27], Hsiao et al. [22], and Lin et al. [28], respectively. In brief, a sample solution with a concentration of 10 mg/mL was produced. A UV-VIS microplate spectrophotometer (Multiskan SkyHigh, Thermo Fisher Scientific, Singapore) was used to quantify the absorbance. Ascorbic acid was employed as the standard. The following formula was used to calculate the percentage of scavenging activity:

Radical scavenging (%) = [(A_blank_ − A_sample_)/A_blank_] × 100 (3)

#### 2.6.2. Total Phenolic Compounds (TPC)

The Follin–Ciocalteu method, described by Gąsecka et al. [27], was utilized to determine TPC. Briefly, 10 mg/mL of sample solution was combined with distilled water, Folin–Ciocalteu’s reagent, and Na_2_CO_3_ solution. At 765 nm, the absorbance was measured. Gallic acid was utilized as the standard. The TPC was calculated as mg gallic acid equivalent per 100 g dry weight sample.

#### 2.6.3. Total Carbohydrate

The total carbohydrate was evaluated by phenol-sulfuric method [29]. Dilute 100× of 10 mg/mL sample solution. A sample was combined with a phenol solution and sulfuric acid. The absorbance at 490 nm was measured. D-glucose was employed as the standard. The data was presented as a percentage of total sugar in the product (g of total carbohydrate in 100 g of the dry weight sample).

### 2.7. Scanning Electron Microscopy (SEM)

SEM (JEOL JSM-7800F Prime Schottky, JEOL USA, Inc., Peabody, MA, USA) was employed to visualize the changes in surface morphology upon extraction of POPs. Each *P. ostreatus* powder sample was attached to the SEM stub with double-sided adhesive carbon tape with a conducting sample holder and sputter-coated with golds under vacuum using a sputtering system [30]. 

### 2.8. Fourier-Transform Infrared (FTIR) Spectroscopy

The alterations in molecular structure upon extraction of POPs were identified using FTIR. Each *P. ostreatus* powder sample was pressed into 1.0 mm pellet. The spectra were recorded by Thermo Nicolet 6700 ATR-FTIR spectroscopy (Thermo Fisher Scientific, Taichung, Taiwan) in the region of 4000–1000 cm^−1^ [31].

### 2.9. Statistical Analysis

Each quality assessment was carried out in triplicate, and the results were averaged and standardized. SPSS statistical software version 26 (SPSS Institute, Chicago, IL, USA) was utilized for the one-way analysis of variance (ANOVA) on the statistically analyzed data. Duncan’s new multiple range test was utilized to determine statistically significant (*p* < 0.05). The optimization was carried out using the RSM (Minitab version 21, LLC, State College, PA, USA). The SigmaPlot statistical analysis software version 12 (Systat Software, San Jose, CA, USA) was used to generate three-dimensional graphs of the obtained models.

## 3. Results and Discussion

### 3.1. Single Factor Analysis

The results of the effects of four single factor parameters (sample-to-water ratio, power, gas flow, and treatment time) of PAW on the yield of POP are shown in Figure 2.

#### 3.1.1. Effect of Sample-to-Water Ratio on Polysaccharide Yield

The effect of sample-to-water ratio at seven distinct levels (1:10, 1:15, 1:20, 1:25, 1:30, 1:40, and 1:50 g/mL) on the percentage of polysaccharide yield was examined in this study. Other parameters were set constantly at 400 W of power, 20 L/min of gas flow, and 3 min of treatment time. It was found that the polysaccharide yield became higher as the sample-to-water ratio increased and reached the maximum yield (11.00 ± 0.13%) when the ratio was 1:20 (Figure 2a), and steadily declined (*p* < 0.05) to 3.32 ± 0.28% in the 1:50 sample. The sample-to-water ratio is one of the key indicators of economic effectiveness for extraction [32]. Generally, a larger sample-to-water ratio could improve the solvent’s diffusivity into cells and expedite the polysaccharides’ desorption from the cells. At the same time, excessive water might absorb energy throughout the extraction process, leading in a decreased extraction yield [33]. Dong et al. [34] emphasized that an elevated ratio of raw material to water diminishes the concentration and viscosity of the extraction solvent, thereby easing the dissolution of polysaccharide molecules in water. This phenomenon might be ascribed to the considerable water-to-material ratio, leading to a notable concentration disparity between the interior of plant cells and the external solvent [35]. Furthermore, a potential contributing factor might be the initial insufficiency of solvent during extraction, causing polysaccharides to quickly saturate after dissolution. Consequently, an excess of solvent could hinder diffusion, resulting in the incomplete leaching of polysaccharides [36]. Additionally, increasing extraction working volume and evaporation energy input is uneconomical [37]. Hence, ratios of sample-to-water from 1:10 to 1:30 g/mL were chosen for the further optimization of polysaccharides extraction. 

#### 3.1.2. Effect of Power on Polysaccharide Yield

The experimental power of 300, 400, 500, 600, 700, and 800 W was implemented in accordance with the PAW machine’s capability, while other extraction conditions were kept constant at 1:20 sample-to-water ratio, 20 L/min of gas flow, and 3 min of treatment time. Figure 2b shows that the polysaccharide yield improved with increasing power (*p* < 0.05), peaking at 10.96 ± 0.47% at 500 W and declining to the lowest (8.83 ± 0.55%) at 700 W. However, the polysaccharide yield increased again when the power was raised to 800 W. Rezaei et al. [38] revealed compatible results where a rise in the extraction yield of fennel seed and spearmint leaf can be observed by increasing the power up to a certain threshold before declining further. The power directly impacts the electric conductivity of PAW and the creation of RONS, which primarily cause cell membrane disruption and improve the important compound release. An adverse effect is that excessively high power supplies undesirably damage the cell [19]. Based on the findings, a power range of 300 to 700 W was chosen to perform optimization of polysaccharides extraction.

#### 3.1.3. Effect of Gas Flow on Polysaccharide Yield

According to the PAW machine capacity, five distinct levels of gas flow rate (15, 20, 25, 30, and 35 L/min) were examined in this study. The following extraction variables were set: 1:20 of sample-to-water ratio, 400 W of power, and 3 min of treatment time. It was discovered that a gas flow of 15 L/min resulted in the highest polysaccharide yield (*p* < 0.05), which was 11.08 ± 0.92%. When the gas flow was raised above 20 L/min, the polysaccharide yield was found to decrease (5.51 ± 1.24 to 7.86 ± 065%), as shown in Figure 2c. Gas flow factors substantially affect gaseous RONS production and solution penetration. Bubbles in submerged orifices may transport RONS from plasma to liquid, may be causing mass transfer mechanisms and promoting PAW activity [39,40]. However, in this study, employing a high gas flow (20–35 L/min) for polysaccharide extraction proved to be not as effective as utilizing a low gas flow (15 L/min). This observation aligns with other research findings [39,40] supporting that the gas flow rate reduces RONS concentrations. The higher gas flow rate, possibly exacerbating the adverse effects, does not align with the requirements of energy and cost efficiency. Consequently, a consistent gas flow of 15 L/min was chosen to optimize polysaccharide extraction.

#### 3.1.4. Effect of Treatment Time on Polysaccharide Yield

The extraction time is an essential factor in the extraction. The PAW extraction time ranging from 30 to 600 s was studied, while other extraction conditions were kept constant at values as follows: sample-to-water ratio, 1:20; power, 400 W; and gas flow, 20 L/min. Figure 2d demonstrates that from 30 to 90 s of plasma treatment, the polysaccharide yield varied from 12.07 ± 0.11 to 12.33 ± 0.29%, which was about 1.2 times greater than the unextracted sample (9.84 ± 0.24%). However, after the plasma treatment reached 120 s, the yield decreased dramatically and continued its downward trend until 600 s, with the lowest yield being 4.16 ± 0.26%, which was less than half that of the untreated sample. Therefore, a PAW treatment time between 30 and 150 s was selected. It might be because plasma can cause the chemical bonds in the starch system to break as a consequence of free radical-mediated depolymerization. Prolonging the plasma treatment time may result in polysaccharide hydrolysis, leading to a decline in polysaccharide yield and a change in microstructure [41,42]. The decrease in polysaccharide yield with prolonged treatment time suggests the potential instability of POPs in water, contrasting with known data on the stability of POPs, which typically include stable components such (1→3)-α-D-glucans, and branched (1→3)(1→6)-β-D-glucans [6]. Various factors may contribute to this observed instability, including potential degradation or breakdown of polysaccharides due to prolonged exposure to water. Extended treatment times could induce hydrolysis or other chemical reactions, leading to a reduction in the polysaccharide yield. Similar trends were noted in polysaccharide extraction from chestnut mushrooms [5] and jelly ear mushrooms [43], where increased extraction time initially improved the yield but continued longer extraction times led to polysaccharide degradation and lower yields. Additionally, Lu, X. [44] provide insights into mushroom polysaccharide stability, suggesting that environmental factors, such as prolonged water exposure or specific temperatures, can differently impact the polysaccharide yield. Understanding the structural features of (1→3)-α-D-glucans and (1→3)(1→6)-β-D-glucans under various conditions further elucidates these observed results. 

The outcomes obtained from the previously mentioned single-factor experiments, a range of ratios 1:10–1:30, power of 300–700 W, treatment time of 30–150 s, and gas flow of 15 L/min were selected to investigate optimization in subsequent response surface analysis.

### 3.2. Optimization of PAW Extraction from P. ostreatus

Based on the results derived from the preliminary screening in single factor analysis, the utilization of the Box–Behnken design for three factors confers some advantage in requiring fewer number of runs; it is also particularly useful when the factors have moderate ranges. As shown in Table 1, the Box–Behnken design recommended a total of 15 experimental runs for the optimization of three extraction variables: PAW power (X_1_), treatment duration (X_2_), and sample-to-water ratio (X_3_). The optimization process was focused on dependent variables, namely the polysaccharide yield (%) and antioxidant activity (%).

#### 3.2.1. Effect of Extraction Variables on Polysaccharide Yield

POPs obtained from the selected range of PAW extraction variables under the Box–Behnken design has a polysaccharide yield in a wide range from 4.55 to 12.17% (Table 1). The independent and dependent variables were fitted using a second-order polynomial equation to the experimental data. The polysaccharide yield regression equation relating coded levels of PAW extraction variables are given below:

% Polysaccharide yield = 9.364 + 2.614X_1_ − 0.731X_2_ + 1.021X_3_ + 0.188X_1_^2^ − 0.615X_2_^2^ − 2.115X_3_^2^ + 0.055X_1_X_2_ + 0.837X_1_X_3_ + 0.231X_2_X_3_ (4)

where X_1_, X_2_, X_3_ were coded values of PAW power, PAW treatment time, and sample-to-water ratio, respectively.

The aftereffects of the ANOVA analysis are shown in Table 2. The model exhibited statistical significance (*p* < 0.0001), implying that the model could be used to predict the optimum condition. Notably, X_1_, X_2_, X_3_, X_3_^2^, and X_1_X_3_ emerged as significant model terms. Positive coefficients for X_1_, X_3_, and X_1_X_3_ in Equation (4) signify a synergistic effect on the POP yield, while negative coefficients for X_2_, and X_3_^2^ denote an antagonistic effect on the POP yield. An evaluation based on the F values revealed that X_1_, X_3_, and X_2_ exerted the largest factor impact on the POP yield. Additionally, the model’s coefficient of determination (R^2^) was 0.8639, suggesting the model is reliable; only 13.61% of aggregate variations was not captured via the model. The lack of fit, judged as non-significance (*p* = 0.069), further substantiated the adequacy of the predicted model.

**Figure 3:**
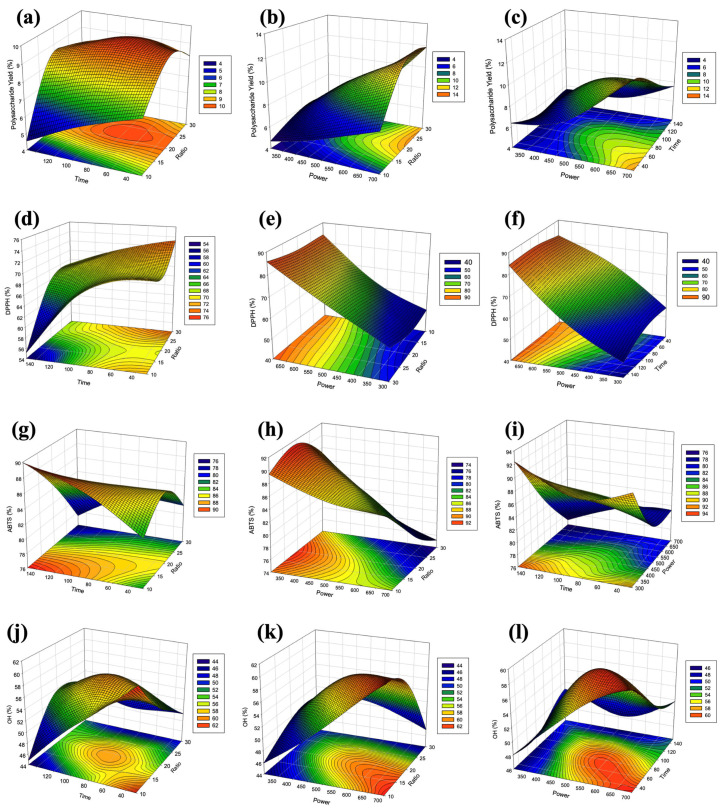
Response surface plot of the POP yield (**a**–**c**), DPPH scavenging activity (**d**–**f**), ABTS scavenging activity (**g**–**i**), and OH scavenging activity (**j**–**l**) with the interaction effects of time and sample-to-water ratio (**left**); power and sample-to-water ratio (**middle**); and power and time (**right**).

For response surface analysis, the polysaccharides yield was determined by the response surface plot and contour plot, both displayed two variables, while maintaining another at a constant value at central level (Figure 3a–c). Notably, an approximately twofold increase in polysaccharide yield was observed with the elevation of the power variable from 300 to 700 W. This observation implies that the heightened ion and electron bombardment led to the etching of the plant cell surface, resulting in rupture and, consequently, an increase in the extraction yield [45]. Similarly, an upward trend in polysaccharide yield was noted by increasing the sample-to-water ratio variable up to 1:20–1:25, reaching a peak and subsequently declining at 1:30. Moreover, a synergistic effect between higher PAW power and higher ratio demonstrated a significant increase in POP yield. In contrast, there was a significant decrease in polysaccharide yield with an increase in PAW treatment time. This aligns with the findings of the single-factor analysis (Figure 2), indicating that prolonged treatment leads to an increased polysaccharide degradation. The model predicted that the maximum polysaccharide yield of 12.71% could be achieved under the optimal conditions as follows: 1:24 ratio, 700 W of power, and 62 s of treatment time. Previous investigations have examined the utilization of cold plasma in oyster mushroom cultivation [46]. Research has revealed that the use of plasma resulted in a notable augmentation of polysaccharide levels. Optimal outcomes were observed with the application of high power and moderate treatment times. In these instances, the heightened energy intensity and plasma radiation generated from the plasma contributed to positive biological effects on cells, promoting polysaccharide productivity.

#### 3.2.2. Effect of Extraction Variables on Antioxidant Activities

Alongside polysaccharide yield, the antioxidant activities were investigated in this study, given that antioxidants are crucial substances for restricting free radicals, preventing oxidation, and preserving food’s nutritional and nutraceutical value. Additionally, employing multiple analytical tests can improve the accuracy of quantification the extract’s or food’s antioxidant potential [1]. The DPPH, ABTS, and OH radical scavenging activities were investigated in this study. 

The fitted model for the scavenging activity of DPPH to predict the relationship between the independent variables and the dependent variable can be expressed with the following formula:

% DPPH = 67.99 + 14.47X_1_ − 3.44X_2_ + 3.82X_3_ − 0.39X_1_^2^ − 0.61X_2_^2^ − 0.84X_3_^2^ + 2.22X_1_X_2_ + 1.94X_1_X_3_ + 2.37X_2_X_3_ (5)

As per the data presented in Table 2, the DPPH model demonstrated statistical significance (*p* < 0.0001). Notably, only the linear variables X_1_, X_2_, and X_3_ were found to be significant. Positive coefficients for X_1_ and X_3_ in Equation (5) indicate a synergistic effect on DPPH, while negative coefficients for X_2_ suggest an antagonistic effect. The factors X_1_ > X_3_ > X_2_ exhibited the most substantial impact on DPPH, respectively. The R^2^ of model was 0.7353 and the lack of fit was found to be non-significant (*p* = 0.458).

Figure 3d–f illustrates the response surface that aligns with the trends observed in the POP yield results, wherein increases in the power and ratio variables corresponds to an increase in DPPH. Conversely, an increase in the PAW time variables coincided with a decrease in the scavenging activity of DPPH. It is possible that more intense plasma treatments could increase the DPPH scavenging activity of *P. ostreatus*. The optimal conditions that provide the maximum DPPH scavenging activity of POPs (87.45%) are a power of 700 W, a duration of 109 s, and a ratio of 1:30.

These findings are in concordance with the study by Rodríguez et al. [47] reported that extended plasma treatments resulted in diminished DPPH antioxidant activities. Regardless of the plasma flow rate, the greatest DPPH antioxidant activity was observed in samples subjected to the shortest treatment duration. On the other hand, Kashfi et al. [48] revealed an increase in the DPPH free radical scavenging percentage of peppermint extract as the power of the plasma increased. The DPPH assay, commonly used for assessing hydrogen donation-based free radical scavenging, has been linked to power-induced modifications in the structure of natural molecules. This modification, particularly affected the double bonds in the carbon or the OH group, resulting in an increase in OH scavenging activity [49]. 

Considering to the ABTS scavenging activity, a second order polynomial equation was proposed:

% ABTS = 85.749 − 5.09X_1_ + 0.173X_2_ − 3.253X_3_ + 0.444X_1_^2^ − 1.033X_2_^2^ − 1.952X_3_^2^ − 1.637X_1_X_2_ − 1.943X_1_X_3_ − 2.781X_2_X_3_ (6)

The model demonstrated statistical significance (*p* < 0.0001), with X_1_, X_3_*,* X_3_^2^*,* X_1_X_2_, X_1_X_3_, and X_2_X_3_ emerging as significant model terms. All significant terms exhibited negative coefficients, indicating antagonistic effect on ABTS. The R^2^ of the model was reliable (0.8598). However, a lack of fit was observed at 0.045, suggesting a significant lack of fit in the model. In practical terms, it may imply the presence of additional factors or complexities in the data that are not accounted for by the current model. Researchers may need to reassess the model, consider additional variables, or explore more complex model structures to better capture the nuances of the data. The response surface findings of ABTS reveal an opposite tendency compared to the polysaccharide yield and DPPH results, where increased power and ratio led to lower ABTS antioxidant capacity (Figure 3g–i). According to the results, the model predicted the optimal conditions for achieving the maximum ABTS of POP (94.20%) to be a power 300 W, time 150 s, and ratio 1:10. Almeida et al. [50] discovered that exposure to higher intensely exposure to plasma resulted in a decrease in ABTS antioxidant capacity, possibly owning to the contribution of oxygen in air plasma, leading to a reduction in antioxidant capacity by producing highly damaging RONS. This phenomenon may cause a decrease in antioxidant capabilities after plasma treatment [1]. 

For estimating the hydroxyl radical (OH) scavenging activity, a second-order polynomial model was developed, with the resulting equation being as follows:

% OH = 58.276 + 3.726X_1_ − 1.867X_2_ − 1.932X_3_ − 2.6X_1_^2^ − 1.882X_2_^2^ − 4.557X_3_^2^ − 1.606X_1_X_2_ − 4.656X_1_X_3_ + 4.786X_2_X_3_ (7)

ANOVA analysis, as presented in Table 2, revealed that the model exhibited statistical significance (*p* < 0.0001). Notably, all linear (X_1_, X_2_, X_3_), quadratic (X_1_^2^, X_2_^2^, X_3_^2^), and interaction (X_1_X_2_, X_1_X_3_, X_2_X_3_) terms emerged as significant model terms. Positive coefficients for X_1_ and X_2_X_3_ in Equation (7) indicate a synergistic effect on the OH scavenging activity, while negative coefficients for X_2_, X_3_, X_1_^2^, X_2_^2^, X_3_^2^, X_1_X_2_, and X_1_X_3_ denote an antagonistic effect on OH scavenging activity. The factors X_1_ > X_3_ > X_2_ exhibited the most substantial impact on OH, respectively. The model achieved a reliable coefficient of determination (R^2^) of 0.8642, while a lack of fit was determined to be significant (*p* = 0.001). 

Figure 3j–l illustrates that an increase in OH scavenging activity is correlated by an increase in PAW power. However, the ratio and time factors showed a contrasting trend, exerting a negative effect on OH scavenging activity. Interestingly, the interaction factor of X_2_X_3_ (time × ratio) demonstrated a greater mutual effect on OH scavenging activity. A shorter PAW treatment time and a lower ratio led to a noteworthy enhancement in OH scavenging activity. The surface plot depicting OH scavenging activity reveals a corner- shift characterized by a more pronounced increase than in the RSM case. Therefore, it is recommended to augment the number of process parameters in future experiments in order to improve the precision of predicting OH scavenging activity in the model. Parameters that do not exhibit significant effects on OH scavenging activity can be excluded or substituted with other parameters to assess their level of significance. The findings of this investigation are congruent with the findings of Hou et al. [51] revealed that prolonged exposure to cold plasma led to decreased OH antioxidant activities in blueberry juice, possibly due to degradation influenced by the higher oxygen concentrations in ionized gas. According to the findings, the optimum approach to achieve the maximum OH scavenging activity (67.81%) is to utilize a power of 700 W, a treatment time of 30 s, and a ratio of 1:10.

It is important to underscore that PAW treatment may not consistently enhance antioxidant capacity and could potentially have an adverse effect. The enhanced antioxidant activity observed could be related to the enzyme inactivation effects of cold plasma, as explained in the literature [52]. Simultaneously, the possibility of plasma-induced oxidation during prolonged treatment times should be considered, highlighting the importance of optimization [53]. Additionally, different PAW process parameters can impact the alteration of antioxidant capacity. Numerous investigations have demonstrated that antioxidants, responsive to a certain approach, can be altered differently through plasma processing, underscoring the importance of employing several analytical assays to precisely identify antioxidant capacity [54,55].

#### 3.2.3. Optimization and Model Validation

In this investigation, we assigned equal weight and importance set to 1 to each response variable to the desirability functions utilized for optimizing the process. The primary goal was to maximize the polysaccharide yield, DPPH scavenging activity, and OH scavenging activity, while explicitly designating ABTS scavenging activity as “do not optimize” due to its behavior exhibiting a divergence from the trends observed in all other responses. The determination of the optimal system point was achieved through the geometric mean maximization of individual desirability functions. Notably, the resulting composite desirability value of 0.9922 holds significance, as it indicates close proximity to the specified targets. The individual desirability values suggest that the settings are more effective for POP yield (d = 1.0000) than for OH scavenging activity (d = 0.99995) and DPPH scavenging activity (d = 0.97672), respectively. This accomplishment reflects the attainment of a POP yield (y = 12.18%), DPPH scavenging activity (y = 84.90%), and OH scavenging activity (y = 61.52%) that align with the predefined requirements. The optimized conditions for POP extraction involve a PAW power of 700 W, PAW treatment time of 58 s, and a sample-to-water ratio of 1:19.

### 3.3. Comparison of Different Extraction Techniques on the POP Yield and the Phytochemical and Antioxidant Activities

**Figure 4:**
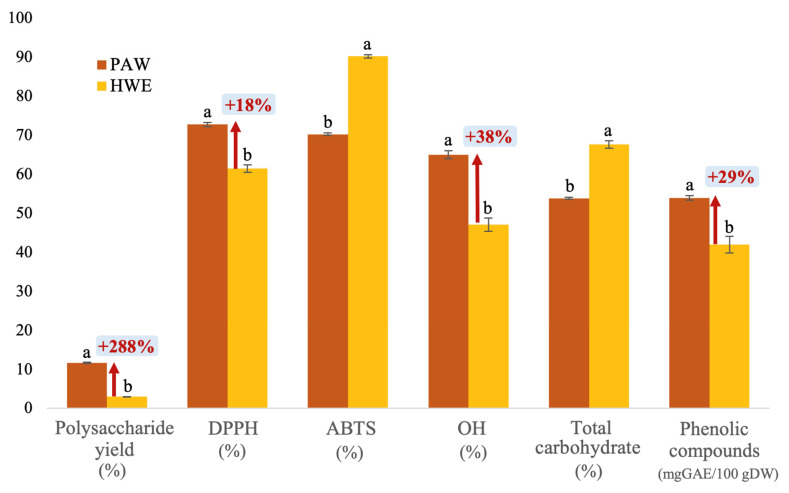
Comparison of different extraction techniques on the physiochemical and biological properties of POPs; the POP obtained by optimized plasma-activated water extraction (PAW) and the POPs obtained by hot water extraction (HWE). ^a,b^ The values with different superscripts exhibit statistical significance (*p* < 0.05). The error bars in the graph represent the standard deviations (*n* = 3).

PAW extraction (700 W, 58 s, and 1:19) was replicated in triplicate in accordance with the previously established optimized conditions. The obtained polysaccharides yield and antioxidant activity were statistically compared (*p* < 0.05) with the values predicted by the model. Additionally, a comparative analysis was conducted with the results of POPs obtained through the hot water extraction technique (100 °C, 3 h, and 1:20). In addition to the investigation and comparison of the polysaccharide yield and antioxidant activity, the phenolic compound and total carbohydrate, deemed crucial values in determining the quality of the extracted polysaccharides, were also investigated and compared, as shown in Figure 4. It was found that the extraction yield, phenolic compound, DPPH, and OH antioxidant of the POPs obtained from PAW extraction were 11.67 ± 0.16%, 53.79 ± 0.61 mgGAE/100 gDW, 72.77 ± 0.53%, and 65.03 ± 1.02%, respectively, which are 288%, 29%, 18%, and 38% greater than hot water extraction, respectively. The direct application of plasma is restricted to a few micrometers under the surface of food, which is controlled by water activity, porosity, and ingredients. Because of the short half-life of ROS such as hydroxyl radicals (1 ns) and singlet oxygen (1 us), effects occur predominantly at the contact surface [56]. The presence of radical-eliminating antioxidants or the occurrence of radical recombination disrupts the chain reaction [57]. The ABTS antioxidant (70.31 ± 0.30%) and total carbohydrate (53.86 ± 0.29%) were 22% and 20%, respectively, lower than the hot water extraction sample. These findings agree with the other authors Kim et al. [49], Bao et al. [58], and Seelarat et al. [59] who reported that the plasma treatment increased the extraction yield of bioactive compounds possessing antioxidant capacity in various plant matrixes, demonstrating a beneficial correlation between phenolic compounds and antioxidant capacity. These increments are due to the existence of conjugated phenolic compounds, which are generally confined to the cellular membrane and cell wall and promptly released when sufficient energy is provided by plasma. An additional reason that may contribute to the elevated solvent penetration is the considerable tissue damage generated by ion bombardment during plasma treatment [1].

On the other hand, ABTS demonstrated the opposite tendency, with a decrease in value after plasma treatment. This is comparable with the results of Almeida et al. [50] and Pogorzelska-Nowicka et al. [60], reported that ABTS is more sensitive, with a 50% reduction compared to the control, while having no effect in other antioxidant assays. It is worth noting that the ABTS method examined a distinct range of processes with other antioxidant methods, and these methods are intricate and encompass multistep reaction mechanisms, making it challenging to find their correlation. The result also showed a decrease in carbohydrate content. Which is supported by Fernandes & Rodrigues [61], who revealed that DBD plasma application decreased carbohydrate content (46%) while increasing phenolic compounds (62%), owing to plasma stress occurring in the phenylpropanoid pathway in food plants, which converts carbs into numerous phenolic compounds. CP treatment can also cause oligosaccharide depolymerization to variable degrees, reducing the average chain length of oligosaccharides. When plasma is applied, ozone oxidation usually leads to depolymerization at α-1,6-glycosidic linkages. 

In summary, the outcomes demonstrated that the PAW technique exhibits the capacity to augment the efficiency and quality of the POP extraction. Compared to conventional hot water extraction which takes three hours longer, the shortened extraction time (less than a min) of PAW extraction outperformed with extraction yield that was three times higher. In addition, without applying high temperatures, PAW extraction also enhances the polysaccharides biological and antioxidant activity. Addressing the challenges in conserving energy and lowering carbon emission are important global issue these days [62]. The extraction of polysaccharides from *P. ostreatus* by plasma-activated water technology is, therefore, an effective approach that is also applicable for extracting other food materials, with possibility of expansion to be used in the food processing companies at larger scales.

### 3.4. Scanning Electron Microscopy

**Figure 5:**
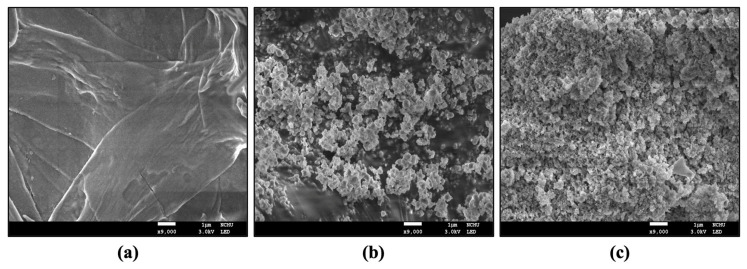
SEM images depicting the powder of unextracted *P. ostreatus* (**a**), the powder of hot water-extracted *P. ostreatus* (100 °C, 3 h, and 1:20) (**b**), and the powder of PAW-extracted *P. ostreatus* (700 W, 58 s, and 1:19) (**c**). A magnification of ×9000 was uniformly applied to all samples.

The shifts in surface characteristics resulting from plasma-induced chemical reactions were described and quantified through SEM, were shown in Figure 5. The findings showed that the unextracted sample had a smooth and intact surface, while a noticeable discrepancy in microstructure was observed in plasma-extracted and hot water-extracted samples. The surface morphology of the sample extracted using hot water disclosed a bilaterally smooth and rough surface with circular particles, while the plasma-extracted sample revealed an obvious roughness with an irregular shape. Furthermore, the presence of pores was discovered in several areas. This observation suggested that the sample’s structure has been degraded further after being treated with PAW. Upon closer inspection, it was noticed that under the same magnification, the plasma-extracted sample displayed a smaller cell size in comparison to the hot water-extracted sample. It is confirmed by the findings of Pogorzelska-Nowicka et al. [60], who discovered that the structures of the plasma-treated sample were greatly damaged compared to the hot water-treated sample. The smaller particle size, higher surface area, and porous appearance can be explained by the ability of plasma etching to disrupt cells [63]. Plasma-induced chemical reactions alter the surface characteristics of materials. Cracks and fragments in the epidermis were induced by electrons and ions produced by plasma reactive species. The occurrence of ruptures leads to an elevation in the polysaccharide granules’ surface energy, thereby enhancing their hydrophilicity and facilitating solvent extraction and modification of the surface microstructure. The present of surface roughness can potentially enhance extraction efficiency by providing more interaction sites between the solvent and the sample may facilitate better penetration, leading to improved accessibility to the polysaccharides. Structural changes accompanying the roughness could improve the yield and quality of the extracted polysaccharides [38,64]. This result provides evidence for the hypothesis that plasma treatment facilitates greater outward release of compounds, thus the extraction effectiveness of the polysaccharides, as well as other bioactive compounds and antioxidant properties, is enhanced. 

### 3.5. FTIR Spectra 

**Figure 6:**
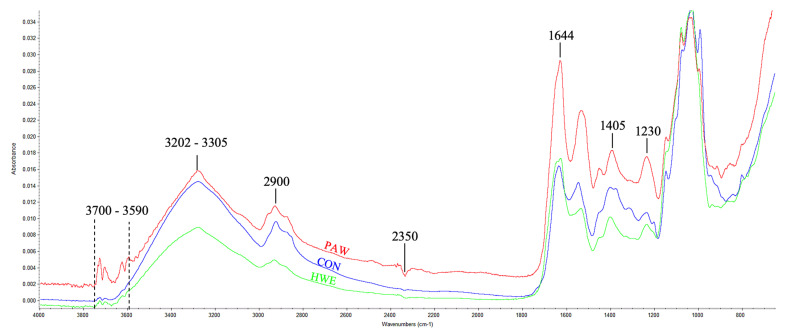
FTIR spectra of an unextracted *P. ostreatus* powder (CON), PAW-extracted POPs (PAW), and hot water-extracted POPs (HWE) in the range of 4000–1000 cm^−1^.

FTIR is used to identify chemical bonds and functional groups on the material’s surface, aiding in the characterization of surface modifications upon extraction of POPs. Figure 6 depicts the FTIR spectra from different samples measured in 4000–1000 cm^−1^ region. It is crucial to note that all of the samples’ FTIR spectra exhibited similarity, with no discernible emergence of new peaks after plasma-treated. The presence of several absorption peaks suggests that the peak at 3700–3590 cm^−1^ is attributed to the free –OH group from alcohol and phenol [65]. The absorption peaks at 3305–3202 cm^−1^ and around 2900 cm^−1^ were attributed to O–H bond stretching in glycosidic bonds and C–H bond stretching on the benzene ring in the polysaccharides, respectively [22,66]. The region of 2350 cm^−1^ corresponds to the O=C=O stretching of carbon dioxide [67], the prominent absorption peak seen at 1644 cm^−1^ is indicative of the presence of the amide C=O bond in the aromatic ring of a polysaccharide–protein complex or protein [68]. Furthermore, the observed peak around 1405 cm^−1^ and 1230 cm^−1^ could be attributed to the O-H vibrations on phenols and the C–O stretching vibrations of the pyranose compounds which are the characteristics of carbohydrates, respectively [22,69]. The finding in this study is consistent with Rashid et al. [64], who observed that cold plasma treatment remained unchanged in the fundamental structure of the sample or induced the formation of new groups of function. 

However, in this study, several band intensities were discovered to be increased in the plasma-treated sample compared to the other samples (Figure 6). For instance, an upsurge in the intensity of the –OH functional group in the range of 3700–3590 cm^−1^, an increase in the intensity of the C=O-O bond (2350 cm^−1^), an increase in the carbonyl bond (1644 cm^−1^), and an increase in the intensity of the C–O bond (1230 cm^−1^). All of those functional groups are hydrophilic due to their polarity which, therefore, leads to an improvement in extraction efficiency of water-soluble POPs which in turn increase the phenolic compounds and antioxidant activities [58,63], according to the findings shown in Figure 4. In addition, it has been shown that plasma has the ability to decrease the pH of the extraction solution by generating HNO_3_, HNO_2_, and H_3_O^+^ ions in the solution when it reacts with RONS. The application of plasma-activated water technology in this study reduces acidic chemical use and environmental impact, hence enhancing the sustainability of the extraction process for POPs [64].

Overall, the findings of this study indicate that the utilization of PAW plasma has the potential to enhance the efficacy of polysaccharide extraction from oyster mushrooms. The author anticipates that the outcomes of this study will have practical implications for the food industry. In particular, the utilization of PAW technology, which has been employed as an alternative to traditional thermal treatments, is expected to contribute to cost and time efficiency and facilitate the sustainable development of novel food products or applied in pharmaceuticals, nutrition, and cosmeceuticals. However, it can be challenging to implement in practice due to the complexity and diversity of technological processes. Further research is required in the areas of mechanistic insights, development of systems, and scalability examinations in order to assess the feasibility of larger-scale commercial applications in the food industry and to assure the safety of consumption [62].

## 4. Conclusions

The experimental design methodology proved to be successful in optimizing the extraction conditions of PAW from POPs. The RSM demonstrated its efficacy in evaluating the impact of three independent variables, namely power, time, and sample-to-water ratio. The most efficient conditions for POP extraction were determined to be 700 W, 58 s, and 1:19 g/mL. Exposure to plasma-activated water was observed to induce surface ruptures and modify the structural properties of materials, enhancing hydrophilicity and resulting in significantly higher yields of polysaccharides, up to approximately 300% compared to conventional hot water extraction methods. The shortening in PAW extraction time, achieved without the application of high temperature, led to an improvement in the biological and antioxidant activities of the obtained polysaccharides. This included a 29% increase in phenolic compounds, a 18% rise in DPPH scavenging activity, and a 38% increase in OH scavenging activity compared to the conventional hot water extraction method. These findings suggest that the extraction of polysaccharides from *P. ostreatus* using PAW technology is both efficient and environmentally friendly. This approach, successfully established as an effective method, warrants further investigation as a novel and potentially valuable natural polysaccharide, facilitating diverse applications within the food industry.

## Figures and Tables

**Table 1:** Box–Behnken experimental design and *P. ostreatus* extraction results.

Run	PAW Condition Parameters			Responses			
	Power, W (X_1_)	Time, s (X_2_)	Ratio, g/mL (X_3_)	Polysaccharide Yield (%)	DPPH (%)	ABTS (%)	OH (%)
1	300	30	20	6.39 ± 0.47	54.21 ± 0.46	90.20 ± 1.24	48.08 ± 0.44
2	300	150	20	5.59 ± 0.27	46.93 ± 1.58	92.34 ± 0.88	50.95 ± 0.25
3	300	90	30	5.63 ± 0.13	55.80 ± 0.75	87.17 ± 0.79	50.03 ± 0.60
4	300	90	10	4.68 ± 0.12	51.62 ± 0.75	89.40 ± 0.79	45.86 ± 0.65
5	500	150	10	4.55 ± 0.40	55.10 ± 2.10	89.88 ± 1.06	44.78 ± 0.60
6	500	90	20	6.86 ± 0.08	47.23 ± 1.87	90.10 ± 0.42	53.13 ± 0.20
7	500	150	30	6.48 ± 0.20	67.06 ± 0.62	77.43 ± 1.04	51.76 ± 0.94
8	500	30	30	8.25 ± 0.37	73.24 ± 0.79	81.21 ± 0.88	49.32 ± 0.94
9	500	90	20	10.27 ± 0.37	77.03 ± 1.05	85.73 ± 0.79	60.93 ± 0.20
10	500	30	10	7.25 ± 0.29	70.75 ± 1.13	82.54 ± 1.30	61.48 ± 0.25
11	500	90	20	10.96 ± 0.77	79.72 ± 0.92	81.42 ± 0.80	60.77 ± 0.43
12	700	90	30	11.87 ± 0.46	85.80 ± 1.80	75.19 ± 1.52	47.07 ± 1.09
13	700	150	20	11.59 ± 0.51	84.20 ± 1.70	77.84 ± 0.64	56.30 ± 0.34
14	700	30	20	12.17 ± 0.18	82.61 ± 1.99	81.21 ± 0.72	59.85 ± 1.00
15	700	90	10	7.57 ± 0.56	83.84 ± 0.90	85.20 ± 0.88	61.52 ± 0.11

**Table 2:** ANOVA of the effect of the PAW extraction variables on POP yield and antioxidant activities.

Source	Polysaccharide Yield (%)				DPPH (%)				ABTS (%)				OH (%)			
	DF	Coef	F-Value	*p*-Value	DF	Coef	F-Value	*p*-Value	DF	Coef	F-Value	*p*-Value	DF	Coef	F-Value	*p*-Value
Model	9		24.68	<0.0001	9		10.80	<0.0001	9		23.85	<0.0001	9		24.75	<0.0001
X_1_	1	2.61	137.79	<0.0001	1	14.74	84.03	<0.0001	1	−5.09	121.07	<0.0001	1	3.73	53.80	<0.0001
X_2_	1	−0.73	10.76	0.002	1	−3.44	4.57	0.040	1	0.17	0.14	0.711	1	−1.87	13.51	0.001
X_3_	1	1.02	21.03	<0.0001	1	3.82	5.66	0.023	1	−3.25	49.47	<0.0001	1	−1.93	14.47	0.001
X_1_^2^	1	0.19	0.33	0.571	1	−0.39	0.03	0.870	1	0.44	0.42	0.519	1	−2.60	12.09	0.001
X_2_^2^	1	−0.62	3.52	0.069	1	−0.61	0.07	0.797	1	−1.03	2.30	0.138	1	−1.88	6.34	0.017
X_3_^2^	1	−2.12	41.62	<0.0001	1	−0.84	0.13	0.725	1	−1.95	8.22	0.007	1	−4.56	37.13	<0.0001
X_1_X_2_	1	0.06	0.03	0.861	1	2.22	0.95	0.336	1	−1.64	6.26	0.017	1	−1.61	5.00	0.032
X_1_X_3_	1	0.84	7.06	0.012	1	1.94	0.73	0.399	1	−1.94	8.82	0.005	1	−4.66	42.00	<0.0001
X_2_X_3_	1	0.23	0.54	0.468	1	2.37	1.08	0.305	1	−2.78	18.07	<0.0001	1	4.79	44.38	<0.0001
Lack-of-Fit	3		2.60	0.069	3		0.89	0.458	3		3.00	0.045	3		7.03	0.001
Constant		9.36				67.99				85.75				58.28		
R^2^		0.86				0.74				0.86				0.86		
R^2^ (adj)		0.83				0.67				0.82				0.83		
R^2^ (pred)		0.81				0.65				0.80				0.80		

## Data Availability

Data is contained within the article.
